# Rapid Genetic Diagnosis of Citrin Deficiency by Multicolor Melting Curve Analysis

**DOI:** 10.3389/fped.2021.654527

**Published:** 2021-05-05

**Authors:** Qinlong Zeng, Yingsong Yang, Jiahong Luo, Jinmei Xu, Choufen Deng, Yuanjuan Yang, Shuming Tan, Shuxiang Sun, Yuping Li, Tong Ou

**Affiliations:** ^1^Medical Genetics Center, Jiangmen Maternity and Child Health Care Hospital, Jiangmen, China; ^2^Department of Pediatrics, Jiangmen Maternity and Child Health Care Hospital, Jiangmen, China; ^3^Prenatal Diagnosis Center and Medical Laboratory, The Third Affiliated Hospital of Shenzhen University (Luohu Hospital Group), Shenzhen, China

**Keywords:** citrin deficiency, SLC25A13, multicolor melting curve analysis, rapid genetic diagnosis, newborn screening

## Abstract

Citrin deficiency caused by *SLC25A13* genetic mutations is an autosomal recessive disease, and four prevalent mutations including c.851_854del, c.1638_1660dup, IVS6+5G>A, and IVS16ins3kb make up >80% of total pathogenic mutations within the Chinese population. However, suitable assays for detection of these mutations have not yet been developed for use in routine clinical practice. In the current study, a real-time PCR-based multicolor melting curve analysis (MMCA) was developed to detect the four prevalent mutations in one closed-tube reaction. The analytical and clinical performances were evaluated using artificial templates and clinical samples. All four mutations in the test samples were accurately genotyped *via* their labeling fluorophores and *Tm* values, and the standard deviations of *Tm* values were indicated to be <0.2°C. The limit of detection was estimated to be 500 diploid human genomes per reaction. The MMCA assay of 5,332 healthy newborns from southern China identified a total of 107 *SLC25A13*-mutation carriers, indicating a carrier rate of 2%. The genotypes of 107 carriers and 112 random non-carriers were validated using direct sequencing and Long-range PCR with 100% concordance. In conclusion, the assay developed in this study may potentially serve as a rapid genetic diagnostic tool for citrin deficiency.

## Introduction

The *SLC25A13* gene is localized on chromosome 7q21.3 and encodes citrin, which is a calcium-stimulated mitochondrial aspartate/glutamate carrier (1). Citrin is expressed abundantly in the liver and serves important roles in urea metabolism, aerobic glycolysis, and gluconeogenesis (2, 3). Citrin deficiency that is caused by biallelic *SLC25A13* mutations is an autosomal recessive disease and can present as neonatal intrahepatic cholestasis (NICCD; OMIM, 605814) in infants or as adult-onset citrullinemia type II (CTLN2; OMIM, 603471) in adolescents and adults (4, 5). Infants with NICCD are characterized by intrahepatic cholestasis, hepatomegaly, diffuse fatty liver, variable liver dysfunction, and hyperammonemia (6, 7). In early infancy, these symptoms overlap with those of other cholestatic liver diseases, such as neonatal hepatitis and biliary atresia, making it difficult for clinicians to obtain a prompt, and accurate diagnosis. Patients with CTLN2 are characterized by exhibiting adult-onset symptoms, hyperammonemia, and a number of neuropsychiatric manifestations (4, 8), and may be misdiagnosed with other neurological diseases. Recently, an additional phenotype, which is characterized by the failure to thrive and dyslipidemia caused by citrin deficiency (FTTDCD), has been described (9). The phenotypic features of patients with citrin deficiency are complex and highly variable. Although, a series of clinical manifestations and biochemical findings have been observed and described in patients with citrin deficiency, none of these features are pathognomonic, and *SLC25A13* genetic analysis has been proposed as an accurate diagnostic tool for citrin deficiency (10–12).

Citrin deficiency was first reported and described in Japan, but later was recognized to be a pan ethnic disease with high prevalence among the East Asian population (1, 11, 13, 14). The overall pathogenic variant carrier rate has been reported to be as high as 2% in southern China, which is the highest carrier frequency currently reported (13, 15). A total of >11,000 individuals are estimated to be homozygous or compound heterozygous for *SLC25A13* pathogenic variants in the Guangdong province in southern China (15). Currently, >80 *SLC25A13* pathogenic variants have been identified, but among them, four prevalent mutations c.851_854del (p.R284fs286X), c.1638_1660dup (p.A554fs570X), IVS6+5G>A (p.A206fs212X), and IVS16ins3kb (p.A584fs585X) account for >80% of the Chinese population who exhibit citrin deficiency (16–18). Therefore, it is important to develop a rapid and cost-effective screening method for these common *SLC25A13* mutations in areas with prevalent citrin deficiency.

A number of methods have been suggested for use in *SLC25A13* genetic analysis, including PCR-restriction fragment length polymorphism, multiplex ligation-dependent probe amplification, mass spectrometry and direct sequencing (10, 19–21). However, the long operational time, low throughput or the requirement of specialized equipment limit the application of these methods in clinical practice. Real-time-PCR-based multicolor melting curve analysis (MMCA) is a recently described detection method that allows the detection of multiple mutations in a single reaction, and has the benefits of speed, ease-of-use and cross-platform compatibility (22). The successful use of MMCA has been reported previously for the detection of genetic disease and pathogen typing (23–25). In this study, a novel MMCA method was developed that allows rapid screening of the four prevalent *SLC25A13* mutations in one closed-tube reaction. The present study also determined this method analytical performance and evaluated its clinical performance.

## Materials and Methods

### DNA Templates and Clinical Samples

For the development and optimization of the MMCA assay, 29 peripheral blood samples with known genotypes (Supplementary Table 1), and eight artificial plasmid DNA templates containing the individual mutations or wild-types of c.851_854del, c.1638_1660dup, IVS6+5G>A, and IVS16ins3kb of *SLC25A13*, were used. The artificial plasmids were prepared by cloning the chemically synthesized gene fragment (the size and sequence of cloned fragment in each plasmid is listed in Supplementary Table 2) into a pUC57 vector, and their sequences were confirmed using Sanger sequencing (Sangon Biotech Co. Ltd). The mixture of c.851_854 wild-type plasmid, c.1638_1660 wild-type plasmid, IVS6+5G>A wild-type plasmid, and IVS16ins3kb wild-type plasmid at a copy-number ratio of 1:1:1:1 was used as an artificial DNA template of the *SLC25A13* wild-type, as well as the other *SLC25A13* genotype plasmid mixtures, respectively (Supplementary Table 2).

For the clinical evaluation and the estimation of the heterozygous carrier frequency, a total of 5,332 healthy newborn blood samples (including 4,574 cord blood, 227 peripheral blood and 531 dried blood spot) were tested. Genomic DNA (gDNA) was extracted from the blood sample using a commercial kit (Tiangen Biotech Co., Ltd.), and the gDNA concentration was determined using a Nanodrop OneC spectrophotometer (Thermo Fisher Scientific, Inc.).

All the clinical samples (29 peripheral blood samples with known genotypes and 5,332 healthy newborn blood samples) were collected from Jiangmen Maternity and Child Health Care Hospital. This study was approved by the Research Ethics Committee of Jiangmen Maternity and Child Health Care Hospital and informed consent was obtained from the participants' legal guardian/next of kin.

### Primers and Probes

DNA sequences of *SLC25A13* (NCBI reference sequence: NC_000007.14) were obtained from the NCBI website (http://www.ncbi.nlm.nih.gov/). To detect the four prevalent *SLC25A13* mutations in the Chinese population, four sets of primers as well as the labeled self-quenched probes targeting the mutation regions were designed using Primer 5.0 software according to the principles of MMCA (Table 1). All DNA oligonucleotides were synthesized and purified by Sangon Biotech Co. Ltd.

**Table 1:** The primer-probe sets used in this study.

**Genotype**	**Primer-probe set**	**Name**	**Sequence**	**Amplicon size**
c.851_854	1	1F	5′-GCCAAACTGAAGGCTATACTG-3′	164 bp
		1R	5′-CTCTTCCAGAGGAGCAAT-3′	
		1P	5′-FAM-TTTGTTTTTCCCCTACAGACGTATGACCTTAGCA-BHQ1-3′	
c.1638_1660dup	2	2F	5′-GACTGAGATGGTGTTGTGT-3′	124 bp
		2R	5′-ACTCCGCTGTAAGTGGTT-3′	
		2P	5′-CY5-ACGAGATTACTGGTGGCTGCCCGGGGAGATTACAG-BHQ2-3′	
IVS6+5G>A	3	3F	5′-CGACTTCCGAGACATCAT-3′	278 bp
		3R	5′-ATTCCGTATTACCCAGACAA-3′	
		3P	5′-CY5-AGTAGCTGTAAGTTGTAAC-BHQ2-3′	
IVS16ins3kb	4	4F	5′-ATACTGCGTGAAGAAGGAC-3′	291 bp (Wild) 447 bp (Mutation)
		4R1	5′-CTACGACAACAGAGCATTAG-3′	
		4R2	5′-CCCTCACTGCTGATTCTTAGATAG-3′	
		4P1	5′-ROX-CAGATTTAGCATGATACTTACACTCCTC-BHQ2-3′	
		4P2	5′-ROX-TTCTTTATATTTGATAGACTGC-BHQ2-3′	

### Real-Time PCR and Melting Curve Analysis

The MMCA assay was performed in a closed tube with 20 μl reaction mixture using a SLAN-48P real-time PCR system (Shanghai Hongshi Medical Technology Co., Ltd.). Each 20 μl PCR reaction mixture contained 1 × PCR buffer (10 mM Tris-HCl; pH 8.9; 50 mM KCl), 0.3 mM dNTPs, 3.75 mM MgCl_2_, 0.5 U Taq HS DNA polymerase (Takara Bio, Inc.), 0.01–0.05 μM limiting primers, 0.07–0.75 μM excess primers, 0.01–0.05 μM probes and 1 μl extracted gDNA or artificial DNA template. The temperature protocol for PCR amplification and subsequent melting curve analysis was as follows: 95°C for 5 min; 10 touchdown PCR cycles of denaturation at 95°C for 30 s, annealing at 60°C for 30 s (–0.5°C/cycle), and extension at 72°C for 30 s; 40 cycles of denaturation at 95°C for 30 s, annealing at 55°C for 30 s and extension at 72°C for 30 s; denaturation at 95°C for 3 min, hybridization at 45°C for 3 min, and a continuous temperature rise from 45 to 80°C at a ramp rate of 0.06°C/s. Fluorescence data from FAM, ROX and CY5 channels were collected at the annealing step during the second 40 cycles and at each step of the continuous temperature rise during the melting stage. Melting curves and fluorescence melting peaks were obtained by plotting the negative derivative of fluorescence over temperature (–dF/dT) vs. temperature. Additionally, graphics output with melting temperature (*Tm*) values were automatically generated in the SLAN-48P real-time PCR system software.

### Analytical Study

Following the establishment of the MMCA assay for detecting the four prevalent mutations, its accuracy, reproducibility and sensitivity were evaluated. To confirm its accuracy, a total of 29 gDNA samples with known genotypes, including 14 wild-type, 11 heterozygous mutant, three homozygous mutant and one compound heterozygous mutant were detected using the MMCA assay in a blinded manner (Supplementary Table 1). To study the reproducibility of *Tm*, a total of five gDNA samples, and six artificial plasmid DNA templates with known genotypes were analyzed by two technicians on 5 non-consecutive days (Supplementary Table 3). The *Tm* values of each genotype were measured and the differences in *Tm* (Δ*Tm*) between the wild-type and mutant were calculated using their mean values. The mean and standard deviation (SD) values of *Tm* values for each genotype were determined using SPSS 22.0 software. To examine the analytical sensitivity of the MMCA assay, all plasmid mixtures for the 15 *SLC25A13* genotypes were serially diluted 10-fold with TE buffer (10 mM Tris-HCl; 1 mM Ethylenediaminetetraacetic acid; PH 8.0) to generate artificial DNA templates containing gene copy numbers ranging from 10^2^ to 10^5^ copies/μl. Each dilution was analyzed in triplicate, and TE buffer was used as a no-template control.

### Clinical Study

For further evaluation of the clinical performance of the assay and for the estimation of the heterozygous carrier frequency in Jiangmen, which is a city in southern China, 5,332 gDNA samples from healthy newborns were screened for the four prevalent *SLC25A13* mutations using the MMCA assay. All detected mutations and 112 randomly selected negative samples were confirmed using direct sequencing and Long-range PCR.

## Results

### Development of the *SLC25A13* Mutation Detection System

**Figure 1:**
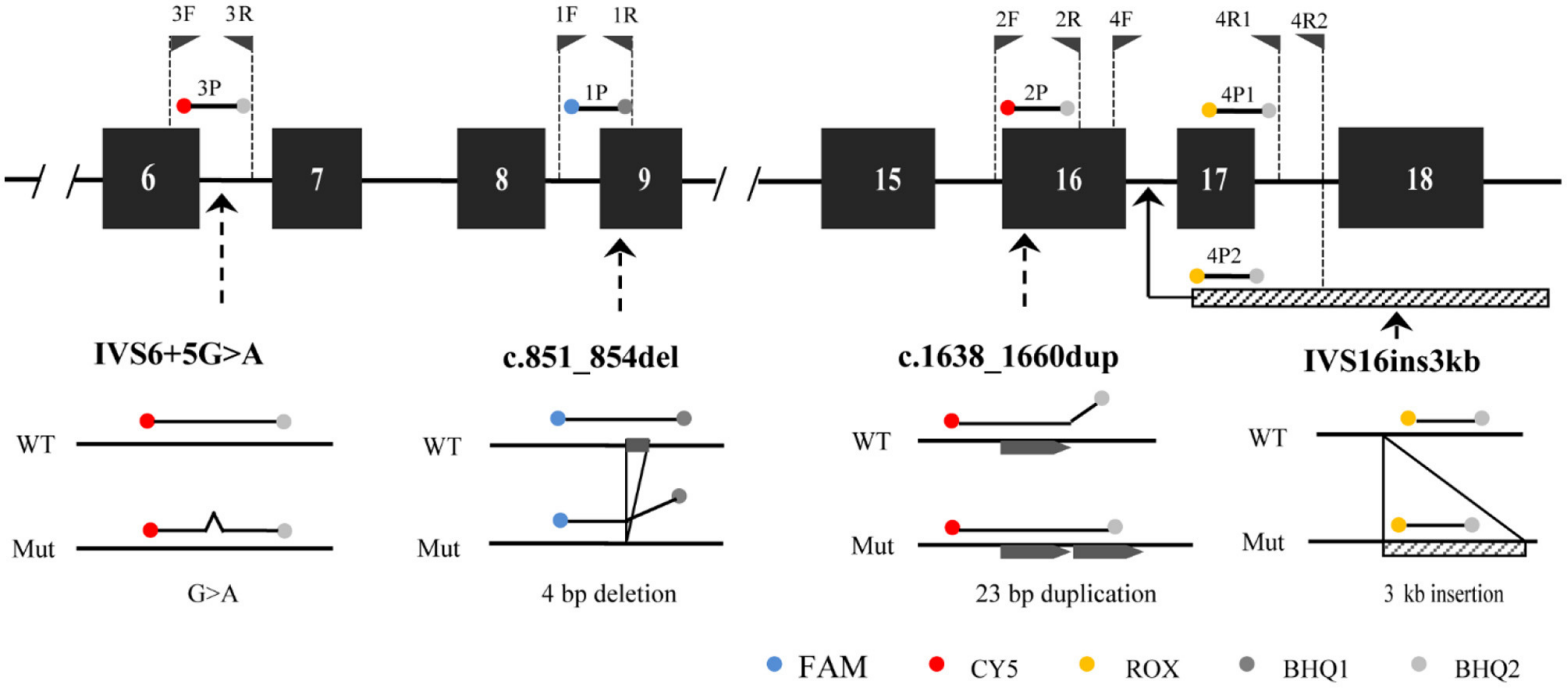
The principle of MMCA assay for the detection of the four prevalent *SLC25A13* mutations. The relative positions of the four designed primer-probe sets within the *SLC25A13* gene are marked. MMCA, multicolor melting curve analysis.

**Figure 2:**
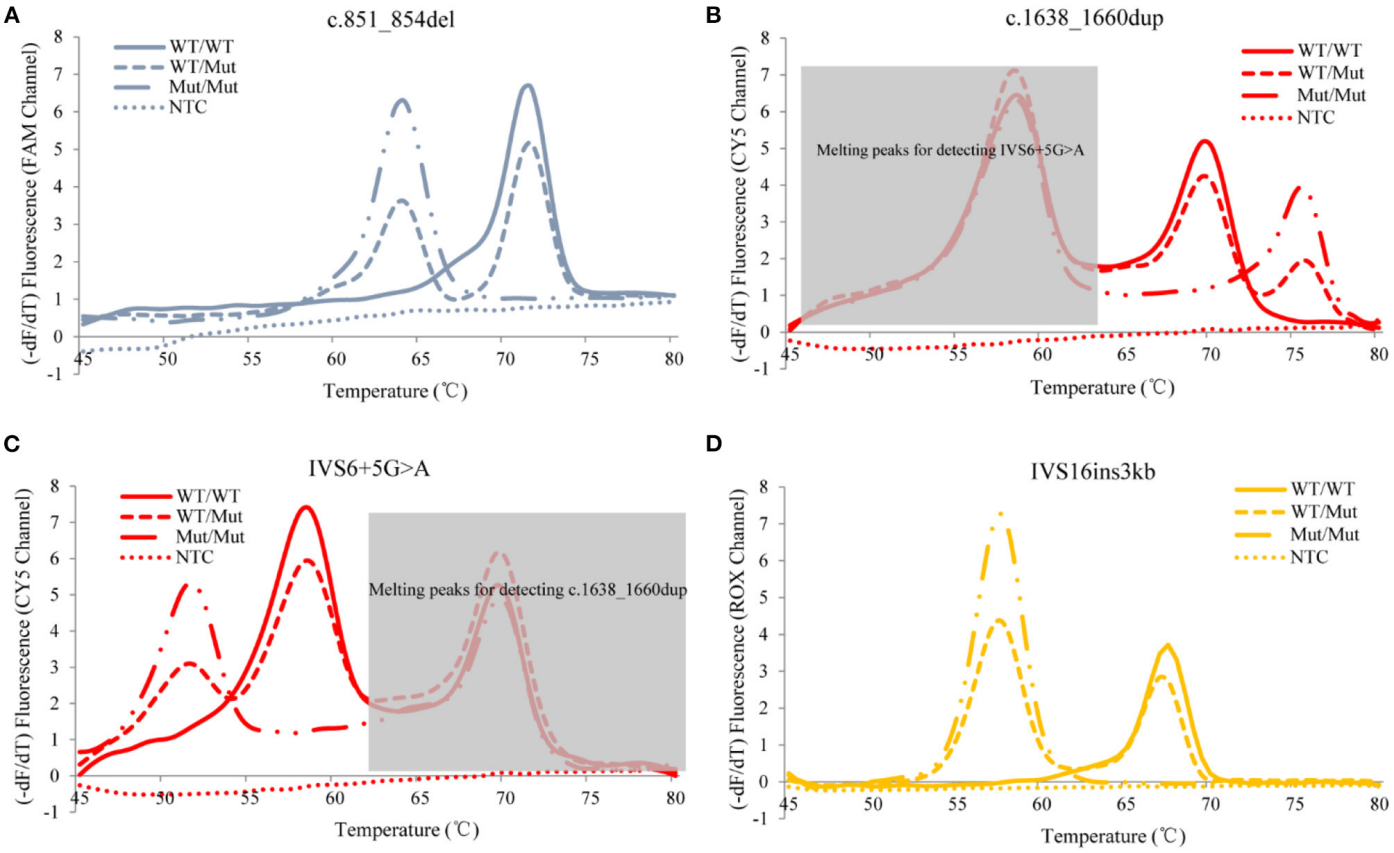
Representative genotyping results of the four prevalent *SLC25A13* mutations using the MMCA assay. Melting curves and fluorescence melting peaks, as well as corresponding genotypes, are provided according to the detection mutations above the panel [**(A)** c.851_854del; **(B)** c.1638_1660dup; **(C)** IVS6+5G>A; **(D)** IVS16ins3kb]. In each panel, melting curves are displayed in only one channel that is related to the detection mutation, and marked on the y-axis. The mutations are individually identified by their labeling fluorophores and *Tm* values, and all mutant alleles generate distinct melting peaks corresponding to the wild-type alleles. –dF/dT, the negative derivative of fluorescence over temperature; WT, wild-type; Mut, mutant; NTC, no DNA template control; MMCA, multicolor melting curve analysis.

The developed *SLC25A13* mutation detection system is a real-time PCR-based multicolor (three color) melting curve assay that detects the four prevalent mutations for Citrin deficiency in one closed-tube reaction using four primer-probe sets. The working principle of this system is illustrated in Figure 1, and the primer-probe sets are presented in Table 1. To genotype the mutations of c.851_854del and IVS6+5G>A, the probes were designed to span the mutation regions and be fully complementary to the wild-type allele in the primer-probe set 1 and 3, respectively. The presence of a four-nucleotide deletion or single-nucleotide substitution would result in a lower *Tm* value compared with that of wild-type allele (Figures 2A,C). To genotype the c.1638_1660dup mutation, the probe in primer-probe set 2 was designed to span the repeat region and be highly complementary to the mutant allele, generating a higher *Tm* value compared with the wild-type allele (Figure 2B). To genotype the IVS16ins3kb mutation, in the primer-probe set 4, a common forward primer and two reverse primers specific to the wild-type and the mutant alleles were used for the amplification of wild-type and insertion mutant alleles, and two probes with different melting temperatures were used for the identification of the amplified allele (Figure 2D). The artificial templates of 15 *SLC25A13* genotypes were detected using the MMCA assay, and the mutations were individually identified by their labeling fluorophores and *Tm* values. All the mutant alleles generated distinct melting peaks corresponding to the wild-type alleles, and the representative results are displayed in Figure 2.

### Analytical Studies

**Figure 3:**
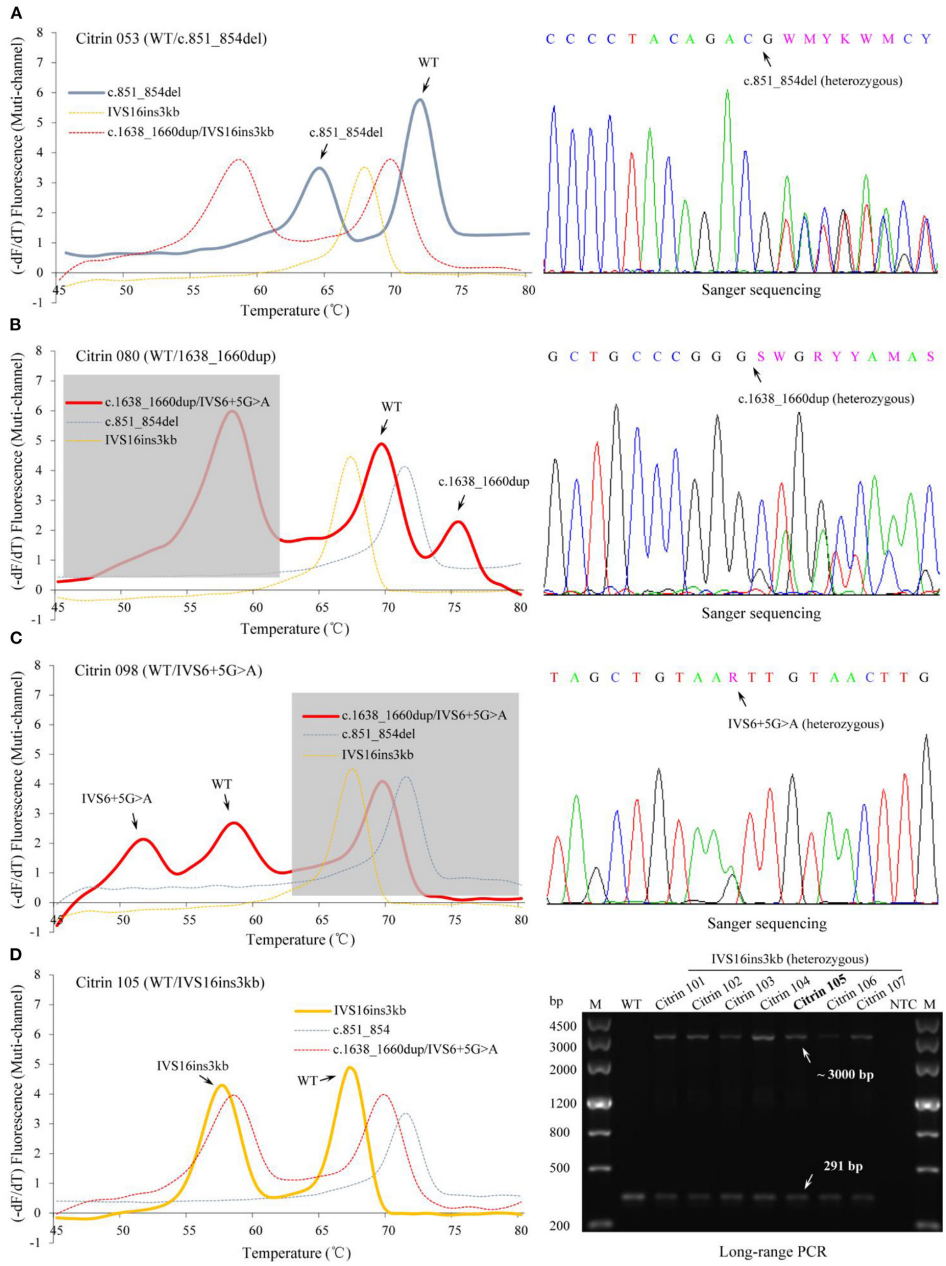
Identification of *SLC25A13* genotypes in the newborn cohort using the MMCA assay. Representative melting curves and fluorescence melting peaks of 4 *SLC25A13*-mutation carriers are presented in the left panels, and the specimen number and genotype of each mutation carrier are marked above the panels [**(A)** Citrin 053, c.851_854del heterozygous carrier; **(B)** Citrin 080, c.1638_1660dup heterozygous carrier; **(C)** Citrin 098, IVS6+5G>A heterozygous carrier; **(D)** Citrin 105, IVS16ins3kb heterozygous carrier]. In each panel, melting curves are displayed in the multi-channel, and the channel revealing the occurrence of the mutation is presented as a solid line. The results of the MMCA assay were further detected by Sanger sequencing and Long-range PCR, obtaining the fully consistent results, which are presented in the right-hand panels. –dF/dT, the negative derivative of fluorescence over temperature; M, marker; WT, wild-type; Citrin 101 to 107, IVS16ins3kb heterozygous carriers; NTC, no DNA template control; MMCA, multicolor melting curve analysis.

Firstly, we evaluated the accuracy of the developed MMCA assay using 29 reference samples with known genotypes, and all the samples gave expected *Tm* values in accordance with the respective genotypes of the four prevalent mutations (Supplementary Table 1). In order to further validate the assays accuracy, the genotypes of 107 *SLC25A13*-mutation carriers and 112 randomly selected non-carriers in 5,332 newborn screenings, which had already been screened using the MMCA assay, were subsequently detected using direct sequencing and Long-range PCR. The genotypes determined using both methods were identical for all 219 gDNA samples, and the representative comparison results from 4 *SLC25A13*-mutation carriers are presented in Figure 3. The MMCA assay accurately detected all the genotypes from the 219 samples, indicating its high sensitivity (100%) and specificity (100%) for the four prevalent *SLC25A13*-mutations. The reproducibility study indicated that the SDs of *Tm* values were <0.2°C in the replicate analysis and all four mutations could be resolved with Δ*Tm* >5°C (Table 2 and Supplementary Table 3). The analytical sensitivity study indicated that all 15 artificial DNA templates revealed reproducible positive results at gene copy numbers ranging from 10^3^ to 10^5^ copies/μl. Therefore, it may be concluded that the MMCA assay exhibited an overall detection limit of 1,000 gene copies per reaction, corresponding to 500 diploid human genomes which is close to 3.5 ng gDNA.

**Table 2:** The *Tm* value of the four prevalent *SLC25A13* mutations in the MMCA Assay.

**Channel**	**Genotype**	***Tm_**1**_*^**°**^C (Mutant) (mean ± SD)**	***Tm_**2**_*^**°**^C (Wild-type) (mean ± SD)**	**Δ*Tm* (*Tm_**2**_-Tm_**1**_*) (mean)**
FAM	c.851_854del	63.86 ± 0.10	71.25 ± 0.07	7.39
CY5	c.1638_1660dup	75.52 ± 0.10	69.70 ± 0.08	***–***5.82
CY5	IVS6+5G>A	51.29 ± 0.15	58.30 ± 0.09	7.01
ROX	IVS16ins3kb	57.45 ± 0.08	67.21 ± 0.08	9.75

### Clinical Evaluation and Frequency of Four Prevalent Mutations

**Figure 4:**
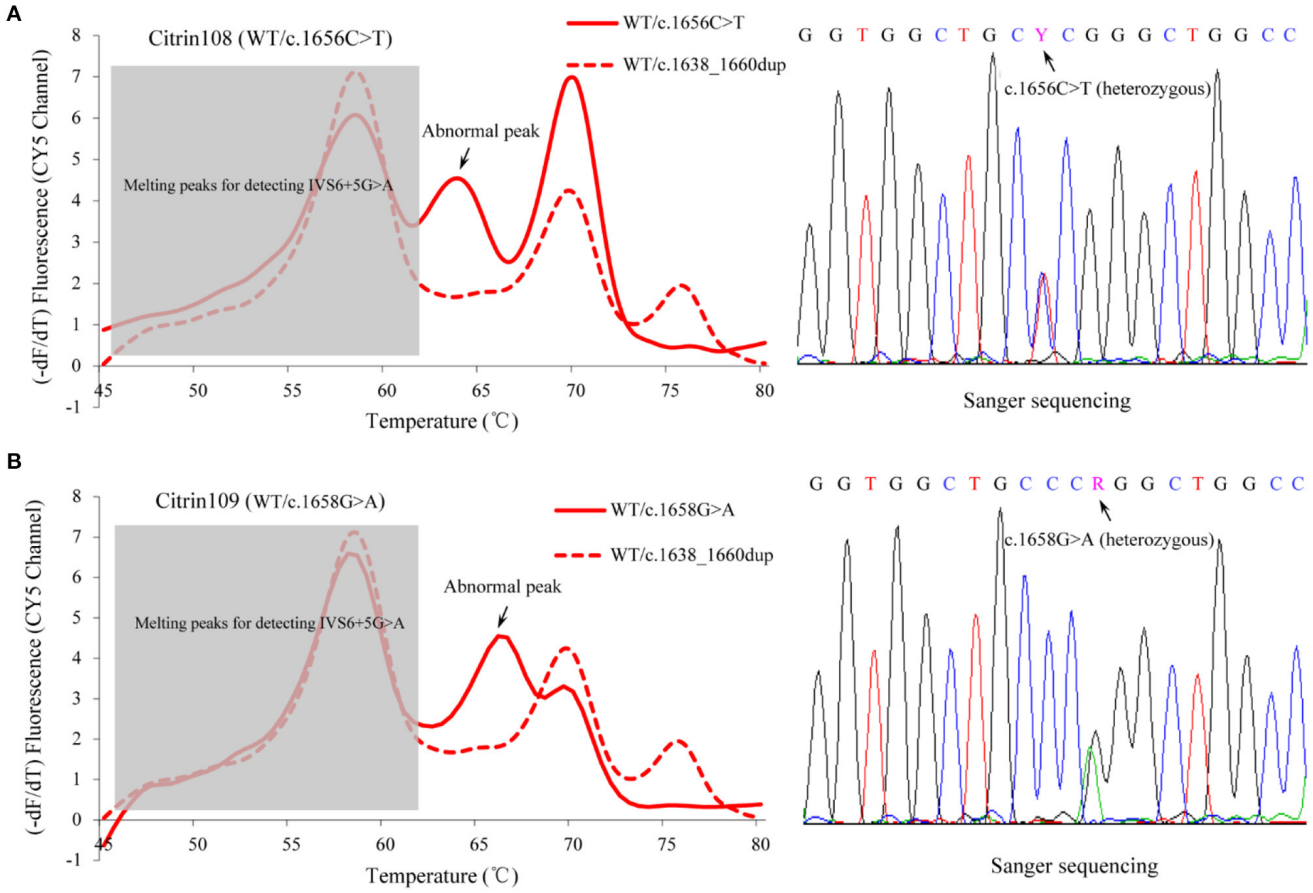
Abnormal fluorescence melting peaks in the MMCA assay. MMCA genotyping of the *SLC25A13* c.1638_1660dup mutation displayed abnormal melting peaks with unexpected melting temperature values in three screening samples, and Sanger sequencing of the probe region uncovered one sample carrying **(A)** heterozygous c.1656C>T mutation and **(B)** two others carrying heterozygous c.1658G>A mutation (one representative is shown). Melting curves are only displayed in the CY5 channel, and the abnormal melting peaks are marked in the solid lines that correspond to the melting curves of the screened samples. The dashed line represents the melting curve of WT/ c.1638_1660dup genotype, indicating the positions of normal melting peaks. –dF/dT, the negative derivative of fluorescence over temperature; WT, wild-type; MMCA, multicolor melting curve analysis.

To further evaluate the clinical performance of the MMCA assay and investigate its potential for screening newborns for citrin deficiency, 5,332 healthy newborns born in Jiangmen were recruited and screened using blood samples remaining from previous clinical testing. The results indicated that 107 newborns were heterozygous for one of the four prevalent mutations, including c.851_854del that was identified in 77 newborns, c.1638_1660dup that was identified in 16 newborns, IVS6+5G>A that was identified in seven newborns and IVS16ins3kb that was identified in seven newborns (Table 3 and Supplementary Table 4). As aforementioned, the parallel Sanger sequencing and Long-range PCR analysis in this cohort indicated fully consistent results. Therefore, the carrier rate of the four prevalent *SLC25A13* mutations was ~2% (107/5,332) in the Jiangmen population. Unexpectedly, MMCA genotyping of the c.1638_1660dup mutation displayed abnormal melting peaks in three screening samples, and Sanger sequencing subsequently uncovered one sample carrying heterozygous c.1656C>T mutation and two samples carrying heterozygous c.1658G>A mutation (Figure 4). Collectively, the MMCA assay can accurately detect the four prevalent *SLC25A13* mutations and simultaneously indicate other possible mutations located in the probe regions.

**Table 3:** The four prevalent *SLC25A13* mutations detected in Jiangmen.

**Genotype**	**Carriers**	**Allele frequency**
c.851_854del	77	1.44% (77/5,332)
c.1638_1660dup	16	0.30% (16/5,332)
IVS6+5G>A	7	0.13% (7/5,332)
IVS16ins3kb	7	0.13% (7/5,332)
In total	107	2.01% (107/5,332)

## Discussion

Citrin deficiency is an autosomal recessive disease that exhibits a number of different clinical manifestations (11, 26). Diagnosis of citrin deficiency requires a high level of clinical suspicion and is confirmed by identifying pathogenic mutations in the *SLC25A13* gene. The *SLC25A13* pathogenic mutation spectrums and the carrier rates present significant geographic differences (13, 27). In southern China, the frequency of *SLC25A13*-mutation carriers was estimated to be 1/48, and the variations c.851_854del, c.1638_1660dup, IVS6+5G>A, and IVS16ins3kb were identified as being the four high-frequency mutations and accounted for >80% of the total mutations in the population (13, 16, 17). In the current study, a novel MMCA assay was successfully developed to detect the four prevalent mutations in one closed-tube reaction. The analytical results indicated that each mutation could be reliably detected from all 29 reference samples and 15 artificial DNA templates. The clinical studies of 107 carrier samples and 112 random negative samples demonstrated that the MMCA assay was as accurate at detecting mutations as direct Sanger sequencing and Long-range PCR.

In the present study, the MMCA assay was performed in a closed-tube PCR reaction, which avoided complex post-PCR manipulations and decreased the risk of PCR contamination. After the gDNA is collected, the entire procedure only requires the addition of gDNA into the reaction tube. In the current study, the cost and turnaround time of this assay was compared with Sanger sequencing and Long-range PCR. The material cost of the MMCA assay for each sample is ~15 CNY and the entire assay can be accomplished within 3.5 h. In comparison, for Sanger sequencing and Long-range PCR, the cost is estimated to be 50 CNY per sample, and the time taken to perform the reaction is doubled. In addition, the high analytical sensitivity of the MMCA assay (3.5 ng/μl gDNA) is beneficial for DNA screening in newborns where dried blood spot samples with limited amounts of extracted DNA are commonly used. In the current study, the minimum concentration of gDNA extraction from 531 dried blood spot samples was 5.9 ng/μl, and all dried blood spot samples were successfully screened for the four prevalent *SLC25A13* mutations. Kikuchi et al. (28) established and reported a real-time PCR-based melting-curve analysis system with adjacent hybridization probes for 11 common *SLC25A13* mutations in the Japanese population. However, in their analysis system, the prevalent mutation IVS6+5G>A was not included, and the other three prevalent mutations were detected in three PCR reactions. In other previous studies, the real-time PCR-high resolution melting analysis was reported to be suitable for screening the presence of *SLC25A13* mutations, but required further sequencing to identify the mutation types (25, 26). Tokuhara et al. (29) reported that citrin was deficient in lymphocytes among patients with citrin deficiency and Western blot analysis of citrin protein in lymphocytes isolated from peripheral blood was established as an alternative diagnostic method for citrin deficiency even in patients without known genetic mutations. Our method, in combination with protein analysis, may have various benefits (e.g., improving the diagnostic yield and clarifying the aetiology) for the diagnosis of citrin deficiency.

A molecular survey on citrin deficiency epidemiology in Guangdong province, China, reported that the carrier rate of the four prevalent *SLC25A13* mutations was ~2.06% (50/2,428), theoretically, with the number of citrin-deficiency patients >11,000 in this population (15). These survey samples were collected from different cities in Guangdong province, but did not include Jiangmen city. The current study collected 5,332 samples from Jiangmen and screened the four prevalent mutations, showing the similar carrier rate of 2.01% (107/5,332). The present study has expanded and detailed the epidemiologic data for the evaluation of citrin deficiency effect in the Guangdong population. The *SLC25A13* mutation spectrum in a large citrin deficiency cohort (274 cases involved 264 Chinese families) indicated that the four prevalent mutations had a relative combined frequency of ~85% (18). These data suggest that the detection of the four prevalent mutations using the MMCA assay could be initially performed for the rapid genetic diagnosis of patients with citrin deficiency in China, and especially in Guangdong. Furthermore, although a diversity of *SLC25A13* pathogenic variants was discovered in the East Asian populations with significant geographic differences, three of the four mutations included in this MMCA assay (c.851_854del, c.1638_1660dup, and IVS16ins3kb) were also common in other East Asian populations, including in Japan and Korea (13, 27, 30). Therefore, the developed MMCA assay may also be adapted and used in other East Asian populations.

In the newborn screening procedure, the heterozygous c.1656C>T mutation and heterozygous c.1658G>A mutation were observed using the MMCA assay and the results were further confirmed by Sanger sequencing. The variant of c.1658G>A causes the change of the amino acid sequence R553Q, which is considered to be a damaging variant and may result in a deleterious effect on protein function as reported in a previous study (31). The variant of c.1656C>T is a synonymous mutation and is not located in the splice site, suggesting that the novel mutation is likely to be a rare polymorphism. Therefore, other possible mutations that are located in the probe regions could also be indicated by the MMCA assay. In addition, to the best of our knowledge, the MMCA assay may be able to detect ≥8 mutations in a one-tube reaction using the real-time PCR instrument with four-color channels. This means that other prevalent *SLC25A13* mutations can be added to the assay to further improve its diagnostic performance for citrin deficiency.

Tandem mass spectrometry (MS/MS) technology provides an opportunity to identify several inherited metabolic diseases in a single test and is widely applied in newborn screening (32, 33). In Italy, expanded newborn screening using MS/MS is currently carried out by law for about 40 inherited metabolic diseases, including citrin deficiency (32, 34). Elevated citrulline values and ratios of citrulline to total amino acids are the primary screening markers for citrin deficiency (21, 35). Because of the instability of citrulline level after birth and the uncertainty about the relation of citrulline level and genotype, it is still challenging to accurately detect citrin deficiency in newborn metabolic screening using MS/MS (21, 35). The combination of MS/MS with genetic approach could aid in improving the detection performance of newborn screening and elucidating the genetic background of citrin deficiency. In addition, the MMCA assay could efficiently identify prevalent *SLC25A13* mutations, which would be a good test for preconception carrier screening and contribute to the prenatal counseling of citrin deficiency.

In conclusion, a closed one-tube MMCA assay for the detection of the four prevalent *SLC25A13* mutations, including c.851_854del, c.1638_1660dup, IVS6+5G>A, and IVS16ins3kb was developed in this study. While the detected mutations are limited at current stage, these mutations account for the majority of the Chinese population with citrin deficiency, and the detection performances were validated in the analytical and clinical studies. This accurate, rapid and cost-effective citrin deficiency genotyping assay could be useful for pre-conception carrier screening and improve the performance of newborn screening by combining metabolic and genetic approach.

## Data Availability Statement

The original contributions presented in the study are included in the article/Supplementary Material, further inquiries can be directed to the corresponding author/s.

## Ethics Statement

The studies involving human participants were reviewed and approved by the Ethics Committee of Jiangmen Maternity and Child Health Care Hospital. Written informed consent to participate in this study was provided by the participants' legal guardian/next of kin.

## Author Contributions

QZ and TO conceived the study and mainly contributed to data analysis and drafting of the manuscript. YL, CD, and YuY provided clinical samples and relevant information. QZ, JX, ST, and SS kept and pretreated samples. QZ, YiY, and JL performed experiments. TO and YL contributed scientific insights and refined the manuscript. All authors reviewed and approved the manuscript.

## Conflict of Interest

The authors declare that the research was conducted in the absence of any commercial or financial relationships that could be construed as a potential conflict of interest.

## Abbreviations

*SLC25A13*: solute carrier family 25 member 13
MMCA: multicolor melting curve analysis
Tm: melting temperature
NICCD: neonatal intrahepatic cholestasis caused by citrin deficiency
CTLN2: adult-onset citrullinemia type II
FTTDCD: failure to thrive and dyslipidemia caused by citrin deficiency
gDNA: genomic DNA
–dF/dT: the negative derivative of fluorescence over temperature
CNY: Chinese Yuan.
